# Targeting the EMT transcription factor Snail overcomes resistance to osimertinib in *EGFR*‐mutant non‐small cell lung cancer

**DOI:** 10.1111/1759-7714.13906

**Published:** 2021-05-04

**Authors:** Qiong Qin, Xiaoqing Li, Xingmei Liang, Lili Zeng, Jing Wang, Linlin Sun, Diansheng Zhong

**Affiliations:** ^1^ Department of Oncology Tianjin Medical University General Hospital Tianjin China; ^2^ Tianjin Lung Cancer Institute Tianjin Medical University General Hospital Tianjin China; ^3^ Phase I clinical trail Department, Tianjin Medical University Cancer Institute and Hospital Tianjin China

**Keywords:** acquired resistance, CDK4/6 inhibitor, epithelial‐mesenchymal transition (EMT), Snail, tyrosine kinase inhibitor (TKI)

## Abstract

**Background:**

The resistance mechanism of the third generation of epidermal growth factor (EGFR) tyrosine kinase inhibitor (TKI) osimertinib is complex. Epithelial mesenchymal transition (EMT) is a common mechanism of EGFR‐TKI acquired resistance. Snail is an important transcription factor related to EMT. Whether targeting Snail can reverse the resistance of osimertinib by downregulating Snail is unknown.

**Methods:**

The presence of EMT in H1975/OR (osimertinib resistance) cells was confirmed by transwell assay. To explore the EMT role in resistance, the expression levels of EMT markers were detected in both parental cells H1975 and resistant cells H1975OR. We used RNA interference technology to knockdown the key regulator Snail in resistant cells. After the interference efficiency was confirmed, changes in EMT‐related molecules of Snail were explicitly downregulated, and changes in sensitivity and migration and invasion ability were also examined. We used CDK4/6 inhibitor to test the ability of reversing drug resistance by downregulating Snail.

**Results:**

Compared with the H1975 cell line, the H1975/OR resistant cell line showed increased invasiveness, upregulated expression of vimentin and downregulation of E‐cadherin. EMT occurred in the H1975/OR resistant cell line. The expression of Snail was upregulated in the osimertinib‐resistant cell line H1975/OR. Knockdown of Snail increased the sensitivity of H1975/OR cells to osimertinib. CDK4/6 inhibitor palbociclib could downregulate the expression of Snail. CDK 4/6 inhibitor palbociclib combined with osimertinib could reverse the resistance of osimertinib in H1975/OR.

**Conclusions:**

Snail plays an important role in the third generation of EGFR‐TKI osimertinib resistance, which may be reversed by downregulating Snail.

## Graphical abstract

In the osimertinib‐resistant cell line H1975/OR, the resistance to Osimertinib and EMT was successfully reversed by downregulating the Snail.

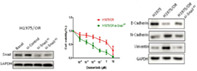

## INTRODUCTION

Lung cancer is the leading cause of cancer‐related death in China and worldwide. Non‐small cell lung cancer (NSCLC) accounts for about 85% of all lung cancer diagnoses.^1^, ^2^ In Asian patients with lung cancer, about 30%–51.4% of lung adenocarcinoma patients have *EGFR* sensitive mutations^3^, ^4^, ^5^, ^6^ sensitive to epidermal growth factor (EGFR) tyrosine kinase inhibitor (TKI) therapy.^7^, ^8^, ^9^However, acquired resistance to EGFR‐TKIs is inevitable. The resistance mechanism of EGFR‐TKIs is complex. The most common resistance mechanism of first generation EGFR‐TKIs is T790M mutation.^10^, ^11^, ^12^, ^13^ In general, third generation EGFR TKI osimertinib has been approved globally for the treatment of T790M‐positive NSCLC patients who have disease progression after therapy with first‐ or second‐generation EGFR‐TKIs.^11^, ^14^ However, it is unfortunate that osimertinib is resistant in the process of continuous use. The mechanism of osimertinib‐resistance is more complex, including C797S, c‐Met amplification, bypass activation, pathological type changes and so on.^13^, ^15^, ^16^ Epithelial mesenchymal transition (EMT) is one of the important mechanisms of EGFR‐TKI resistance.^17^, ^18^, ^19^, ^20^ Previous studies have shown that EMT is one of the important mechanisms of resistance to erlotinib, gefitinib in the first generation and osimertinib third generation of EGFR‐TKIs.^17^, ^19^, ^20^, ^21^ Epithelial‐mesenchymal transition (EMT), manifesting as a loss of E‐cadherin, and an increase in vimentin expression, is a process in which epithelial cells lose their polarity and adhesion to gain migratory ability and adopt a mesenchymal phenotype.^22^ E‐cadherin expression is transcriptionally repressed by Zeb, Snail, Slug, and Twist.^23^ These EMT transcriptional regulators have been suggested as potential therapeutic targets for human cancers.^24^ For example, the expression of Twist has been shown to be increased in an EGFR‐TKI resistant cell line, involved in the regulation of EMT and genetic and pharmacological inhibition of TWIST resulted in growth inhibition and apoptosis in *EGFR*‐mutant NSCLC cell lines.^21^ Knockdown of Hakai has also been reported to elevate E‐cadherin expression, attenuate stemness, and resensitize cells to gefitinib, and dual HDAC and HMGR inhibitor JMF3086 inhibited the Src/Hakai and Hakai/E‐cadherin interaction to reverse E‐cadherin expression, and attenuated vimentin and stemness to restore gefitinib sensitivity.^19^, ^21^

However, Snail is a key transcription factor of EMT, which has been found to promote the transformation of tumor epithelial cells into stromal cells, enhance the movement and invasion ability of tumor cells, promote the dryness of tumor cells and cell migration.^25^, ^26^ In our study, we demonstrated that genetic silencing of Snail or the downregulation of the expression of Snail by CDK4/6 inhibitor palbociclib in EGFR TKI resistant *EGFR*‐mutated cells increased sensitivity to EGFR‐TKI. At present, there is no specific inhibitor of Snail which has been approved by the FDA and CFDA. A previous study reported that CDK4/6 inhibitor palbociclib could regulate Snail expression, inhibit EMT and reduce the occurrence of distant metastasis in triple negative breast cancer.^27^ The main mechanism might be that CDK4/6‐mediated activation of DUB3 is essential to deubiquitinate and stabilize Snail. DUB3 may also promote the proliferation of NSCLC by inhibiting Cyclin A degradation.^28^ Therefore, in our study we used palbociclib to explore its effect on Snail expression and to ascertain whether it could reverse drug resistance.

## METHODS

### Cell lines and cell culture

Human lung adenocarcinoma cell lines H1975 (sensitivity to osimertinib, with a compound *EGFR* exon 21 L858R and T790M mutation) and H1975OR (osimertinib resistance) were used in this study. The NCI‐H1975 human lung adenocarcinoma cell line was obtained from the American Type Culture Collection (ATCC). The osimertinib‐resistant H1975 (H1975OR) cell line was established by our laboratory as previously described.^29^ Cells were cultured in RPMI‐1640 medium supplemented with 10% fetal bovine serum (Gibco) at 37°C in a humidified incubator with 5% CO_2_.

### Cell proliferation assay

The cells H1975 and H1975OR were seeded at a density of 1.0 × 10^4^ cells/well in a 96‐well plate for 48, and 72 hours. Cell counting kit‐8 (CCK‐8) solution (10 μl, B34302, Selleck Chemicals) was added to each well prior to the endpoint of incubation. The absorbance, which represents the cell count, was determined with a microculture plate reader at 450 nm.

### Cell migration assays

Transwell assays were performed using polyethylene terephthalate transwell filters (Millipore; 8.0 μm pore size) placed over a bottom chamber containing RPMI medium with 10% FBS. Cells suspended in serum‐free RPMI medium were added to the upper chamber at the indicated density. After 6–8 h of incubation at 37°C, cells that had migrated to the lower side of the filter were stained with crystal violet. Five fields per transwell were photographed using a microscope at 200× magnification. For wound‐healing assays, cells were seeded in a six‐well plate, and a wound area was generated by scraping with a 1–2 ml pipette tip. After 42 h of incubation, the wounded monolayer was photographed.

### Flow cytometry analysis of apoptosis

A PE‐Annexin V/7‐AAD apoptosis detection kit was used to evaluate apoptosis. Briefly, for apoptosis analysis, cells were cultured 48 h and then collected and stained with PEAnnexin V and 7‐AAD for 15 min in the dark. The cells were analyzed by flow cytometry (BD Biosciences) within 1 h. The data were analyzed with FlowJo software.

### Reverse transcription and quantitative real‐time PCR

Total RNA was isolated using Trizol (ThermoFisher Scientific) and an RNeasy Mini Kit (Qiagen), following the manufacturer's instructions. cDNA was synthesized using the M‐MLV Reverse Transcriptase Kit (Promega) according to the manufacturer's protocol. Quantitative real‐time PCR analysis was performed with ABI SYBR Green Master Mix (Thermofisher Scientific) in an ABI7500 Real‐time PCR System according to the manufacturer's protocol. Each sample was run in triplicate for each gene. Transcript levels were normalized to the housekeeping gene GAPDH and analyzed by the relative quantification 2‐ΔΔCt method. All gene primers were obtained from SBS. We used Affymetrix GeneChip probe to test the gene changes in the RNA of H1975 and H1975/OR cells. All the detected gene primers are shown in Table 1.

**TABLE 1:** The detected gene primers

Gene name	Forward prime(5′ to 3′)	Reverse prime (5′ to 3′)
GAPDH	GGAGTCAACGGATTTGGTCG	CTTGATTTTGGAGGGATCTCG
E‐cadherin	TGA AAAGAGAGTGGAAGTGTCCGAG	GATTAGGGCTGTGTACGTGCTGTTC
N‐cadherin	CAATCCTCCAGA GTTTACTGCCATG	GATTGGTTTGACCACGGTGACTAAC
Vimentin	GCA AAGCAGGAGTCCACTGAGTACC	TGTCAAGGGCCATCTTAACATTGA G
Snail	TCGGAAGCCTAACTACAGCGA	AGATGAGCATTGGCAGCGAG
DUB3	CTATCATTGCGGTCTTTGTCTCC	AAGTGATGCTACAGGCAGTGA

### Western blotting

Protein samples were resolved by sodium dodecyl sulfatepolyacrylamide gel electrophoresis and transferred to polyvinylidene fluoride membranes (Merck Millipore). Membranes were blocked with fat‐free milk combined with tris‐buffered saline plus tween 20 for 1 h at room temperature and then incubated with the appropriate primary antibody and horseradish peroxidaseconjugated secondary antibodies. Imager was used to visualize the blots. The primary antibodies used in this study are as follows: anti‐phospho‐EGFR(Try 1068) (#3777, 1:1000, cell signaling), E‐cadherin (#3195, 1:1000, cell signaling), anti‐vimentin (#571, 1:1000, cell signaling), anti‐Snail (#3879, 1:1000, cell signaling), anti Twist1 (#46702, 1:1000, cell signaling), MMP2 (#40994, 1:1000, cell signaling); MMP9 (#13667, 1:1000, cell signaling); and β‐actin (ab92552, 1:1000).

### Generation of Snail‐knockdown cells by transfecting plasmid

Development of transfection vectors and transfection of H1975/OR cells were performed as standard protocols, with some modifications.^30^ In brief, H1975/OR cells were transduced with plasmid that expressed scrambled siRNA (control, forward: GCACAACAA GCCGAATACA, reverse: UGUAUUCGGCUUUGUUGUGC), Snail siRNA#1 (Forward:: GGACUUUGAUGAAGACCAU, reverse: AUGGUCUUCAUCAAAGUCC), Snail siRNA #2. (forward: GCGAGCUGCAGGACUCUAA, reverse: UUAGAGUCCUGCAGCUCGC). Cells were then washed.

with PBS and cultured before screening for gene expression. Once decreased expression of Snail was confirmed, the cells were used for experiments.

### Statistical analysis

All data were analyzed using GraphPad Prism 7.0 (GraphPad Software). Independent samples *t*‐test were used to identify the statistical significance between groups. ANOVAs were used for analysis of more than two groups of data in all experiments. Significant differences between the means were considered. *p* < 0.05 indicated statistical significance.

## RESULTS

### Invasion of H1975OR cell line increased and apoptosis decreased indicating EMT

**FIGURE 1:**
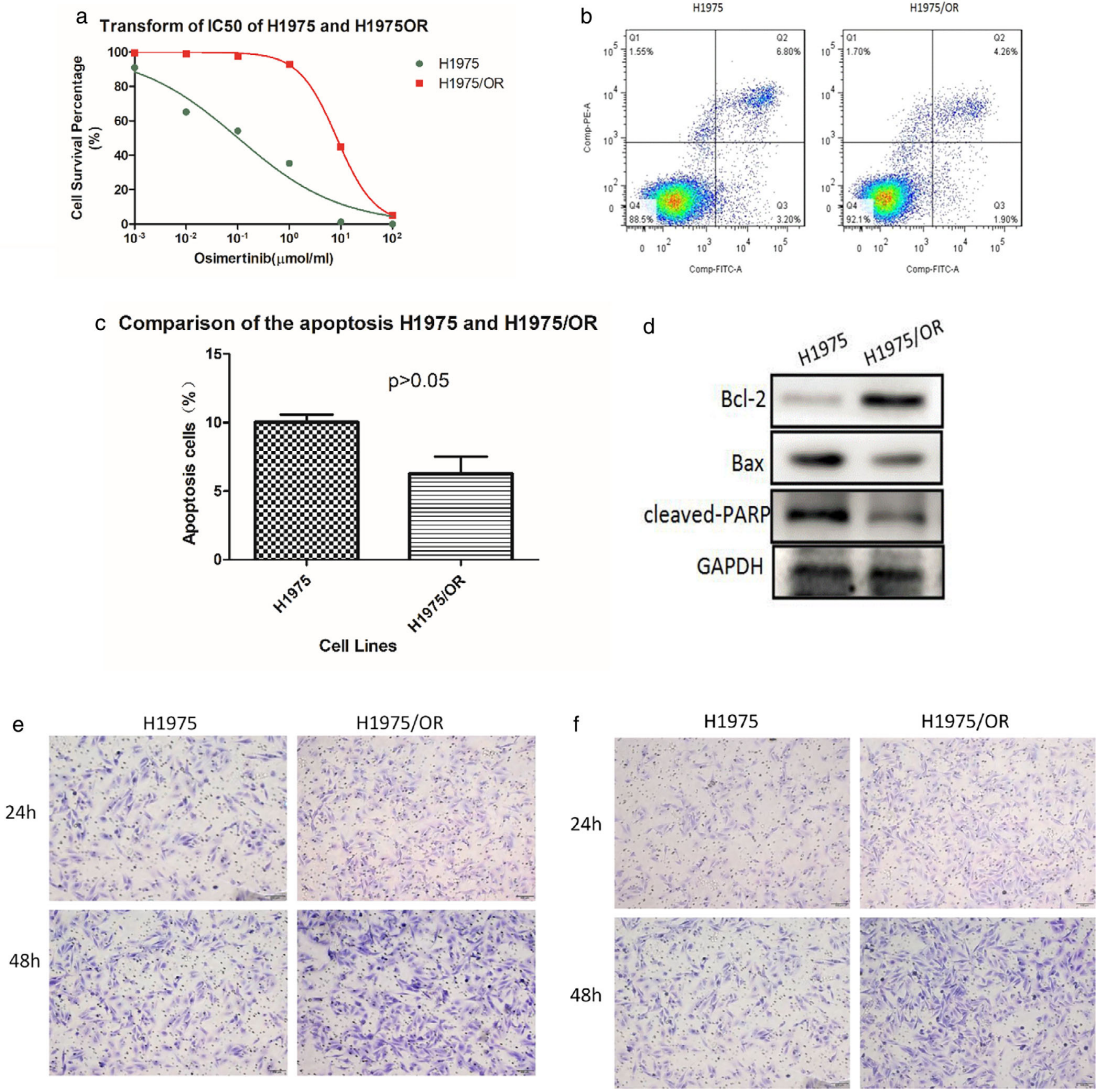
(a) IC50 of H1975 and H1975/OR. (b, c, d) Difference of apoptotic ability between H1975 and H1975OR. (e, f) The invasive ability of H1975 and H1975/OR by transwell assay

The inhibitory dose (IC50) of H1975OR cells was significantly higher than H1975. The IC50 of the two groups of cell lines was 0.1024 and 8.391 μM, respectively (Figure 1(a), *p* < 0.01). The drug resistance index was 81.94, which was much higher than 10, indicating that the cell line was resistant. The proportion of apoptotic cells in parent cell line H1975 was about 10%, and 6.1% in the H1975/OR cell line by flow cytometry (Figure 1(b)), indicating no statistically significant difference (*p* = 0.170, Figure 1(c)). Compared with H1975, the proapoptotic proteins Bax and cleaved PARP were decreased, and the expression of antiapoptotic protein Bcl‐2 was increased in H1975/OR (Figure 1(d)). Transwell migration test was used to compare the invasive ability of H1975 and H1975/OR cell lines. In 24 and 48 h, more H1975/OR cells crossed the transwell, indicating that the H1975/OR cell line was more invasive (Figure 1(e)). Similarly, the transwell invasion test confirmed that the number of H1975OR crossing matrix gel and transwell was more invasive (Figure 1(f)).

### EMT related mRNA and protein in H1975OR

**FIGURE 2:**
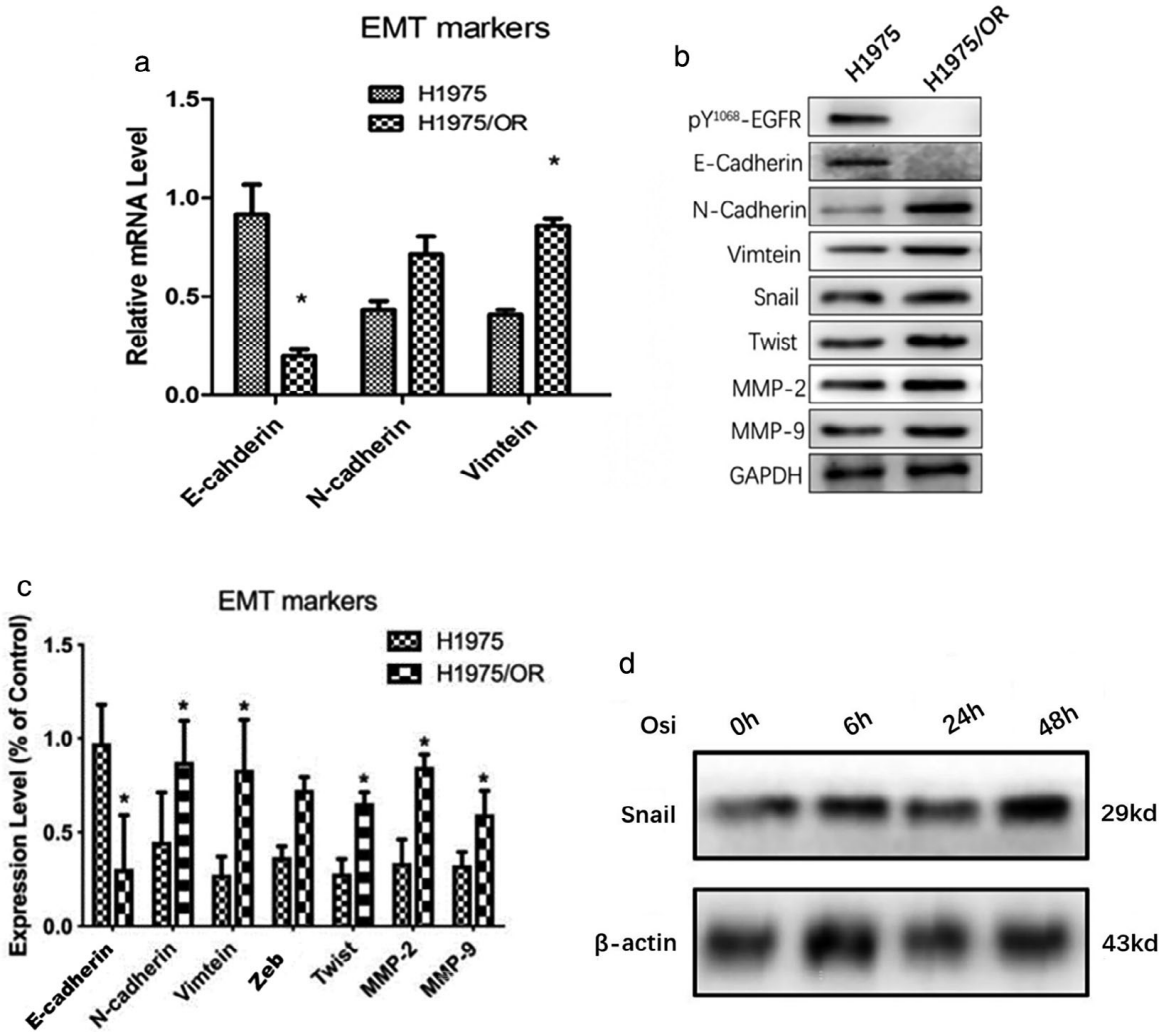
EMT related gene and protein in H1975/OR and H1975 ((a) The level of mRNA of N‐cadherin and vimentin was upregulated and E‐cadherin downregulated. (b) The protein of N‐cadherin, vimentin, Snail and Twist were upregulated and E‐cadherin downregulated. (c) The mRNA of E‐cadherin, N‐cadherin, vimtein, Zeb, Twist, MMP‐2 and MMP‐9. (d) The expression of Snail in H1975 in osimertinib 100 nM)

Compared with the H1975 cell line, N‐cadherin and vimentin mRNA were upregulated and E‐cadherin was downregulated in H1975/OR (Figure 2(a)). Western blot further confirmed that E‐cadherin expression was downregulated in H1975OR, while the expression of N‐cadherin and vimentin was upregulated. MMP‐2, which was related to the invasion ability of tumor cells, was downregulated. The expression of MMP‐9, Snail and Twist were upregulated (Figure 2(b)). In the sensitive cell line H1975, the expression of Snail gradually increased with the continuous application of osimertinib, suggesting that Snail might play an important role in the process of osimertinib resistance (Figure 2(c)).

### Knockdown of Snail reversed osimertinib‐resistance in H1975/OR

**FIGURE 3:**
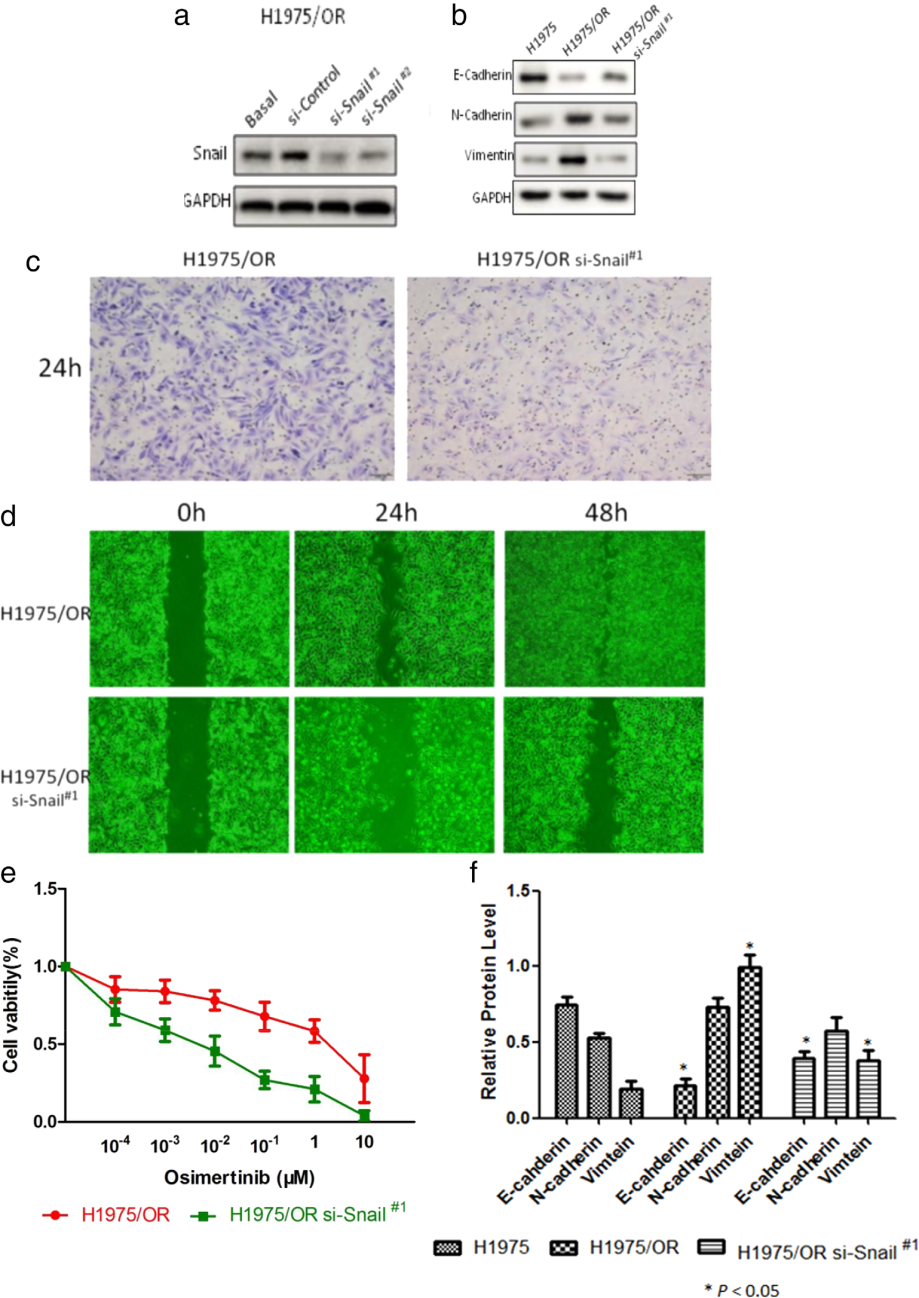
Knockdown of Snail in H1975/OR. (a) The knockdown of snail was verified by Western blot in H1975OR si‐snail^#1^ and si‐snail^#2^. (b) E‐cadherin was upregulated, while N‐cadherin and vimentin were downregulated in H1975OR si‐snail^#1^. (c) The number of cells penetrating the transwell was reduced in H1975OR si‐snail^#1^. (d) Wound healing was slow in H1975/OR si‐snail^#1^. (e) IC 50 of osimertinib was 11.5 nM in H1975OR si‐snail^#1^ (f) mRNA of EMT gene in H1975, H1975/OR and H1975/OR si‐snail^#1^

The expression of Snail was interfered by transfecting plasmid siRNA in H1975/OR. The knockdown of Snail was verified by Western blot (Figure 3(a)). In H1975/OR si‐snail^#1^, the expression of E‐cadherin was upregulated, while N‐cadherin and vimentin were downregulated (Figure 3(b)). In H1975/OR si‐snail^#1^, the number of cells penetrating the transwell was reduced (Figure 3(c)), and the wound healing was slow (Figure 3(d)), which indicated the decline of migration and invasion ability. Compared with H1975/OR, H1975/OR si‐snail^#1^ was more sensitive to osimertinib (Figure 3(e)) and was consistent with the change in EMT (Figure 3(f)).

### CDK4/6 inhibitor palbociclib regulated Snail and reversed osimertinib‐resistance

**FIGURE 4:**
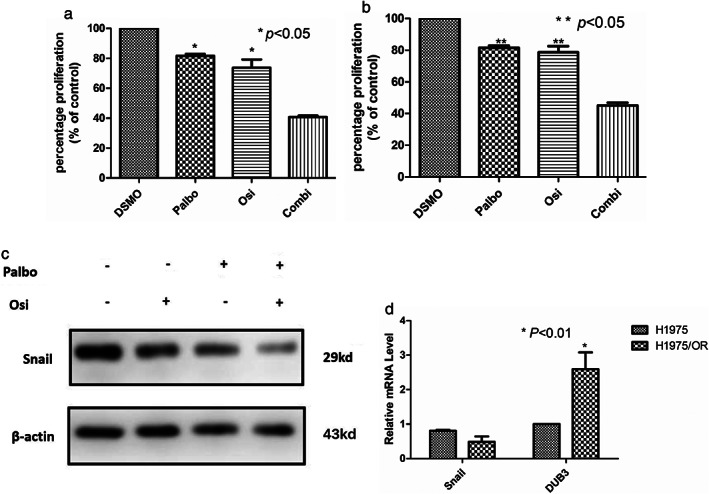
CDK4/6 inhibitor palbociclib combined with osimertinib restrained H1975OR and downregulated Snail. (a, b) Osimertinib (2.5 and 1.25 μM) combined CDK4/6 inhibitor palbociclib 1.25 μM increased inhibition of H1975OR. (c) Osimertinib (2.5 μM) and combined CDK4/6 inhibitor palbociclib (1.25 μM) downregulated Snail. (d) The mRNA of Snail and DUB3 in H1975 and H1975OR

We then determined whether cell cycle arrest with the CDK4/6 inhibitor palbociclib indeed restored the sensitivity of osimertinib. CDK4/6 inhibitor palbociclib at the tested concentration ranges did not or only weakly suppressed the growth of H1975/OR. As expected, H1975/OR was resistant to osimertinib. However, the combination of palbociclib and osimertinib very effectively inhibited the growth of H1975/OR (Figure 4(a),(b)). The difference was statistically significant (*p* < 0.05). In the resistant cell line H1975OR, palbociclib combined with osimertinib could significantly downregulate Snail expression (Figure 4(c)). Furthermore, there were no statistically significant difference in the mRNA level of Snail in H1975 and H1975OR. However, the mRNA of DUB3 in H1975OR was higher than H1975 (Figure 4(d)).

## DISCUSSION

EGFR‐TKI resistance is the main cause of failure in the treatment of patients with *EGFR* sensitive mutations. With the wide application of the third generation EGFR‐TKI osimertinib, more and more patients develop osimertinib‐resistance, which are not T790M. EMT might be one of the important mechanisms of the third generation EGFR‐TKI osimertinib‐resistance. In our study, we confirmed that, CDK4/6 inhibitor palbociclib combined with osimertinib could reverse osimertinib resistance by regulating Snail in the H1975OR cell line.

The expression of Snail in lung cancer is related to DFS and OS in NSCLC. High Snail expression has been reported to be a significant prognostic predictor in patients with pathological N0 NSCLC.^31^ In lung cancer, UGDH promotes tumor metastasis by increasing the stability of Snail mRNA.^32^ In our study, knockdown of Snail reversed the EMT phenotype, downregulated N‐cadherin and vimentin, upregulated E‐cadherin, and reduced the invasion and migration ability of H1975OR. Snail may therefore be a therapeutic target for reversion of EGFR‐TKI resistance.

Traditionally, Snail is considered to be difficult to become a drug target. The drugs which regulate the Snail upstream pathway and indirectly downregulate Snail expression have become the focus of research in the field. This study found that in H1975OR, CDK4/6 inhibitor might downregulate DUB3 by inhibiting the function of CDK4, and Snail may be ubiquitinated and degraded due to the loss of DUB3 protection, thus reducing tumor invasion and metastasis. Further studies are needed to clarify the possible mechanism.

In addition, Li et al. found that CYD19, a new synthetic compound, directly combined with the conserved 174 arginine (r174) in Snail evolution could significantly inhibit Snail protein expression in a variety of tumor cell lines at the level of nM. It mainly interfered with the binding of acetyltransferase CBP / P300 with Snail through the binding of CYD19 with Snail protein, which resulted in the loss of acetylation protection of the latter ubiquitination degradation.^33^

Unfortunately, these studies were only performed in H1975OR, and not in more EGFR‐TKI resistant cell lines. In future, it is hoped that we can further verify these results in more EGFR‐TKI cell lines and a PDX model.

In conclusion, we believe that targeting EMT related transcription factor Snail is an important mechanism for reversing the resistance of EGFR‐TKI to osimertinib in the future. It is expected that clinical trials in more new drugs, such as CYD19, will soon be completed confirming its effectiveness in the human body. At the same time, it is also hoped that CDK4/6 inhibitors could be explored more in EGFR‐TKI‐resistant cell lines in order to provide more support for the combined application of CDK4/6 inhibitors palbociclib and osimertinib.
