# Correction to targeting the EMT transcription factor snail overcomes resistance to osimertinib in EGFR‐mutant non‐small cell lung cancer

**DOI:** 10.1111/1759-7714.14821

**Published:** 2023-02-16

In Qiong Qin et al.^1^ the following error was published on page 1710.

In figure 1, the images on labels e and f were duplicated. The images on label e must be deleted. Figure 1 should show as below:

**FIGURE 1:**
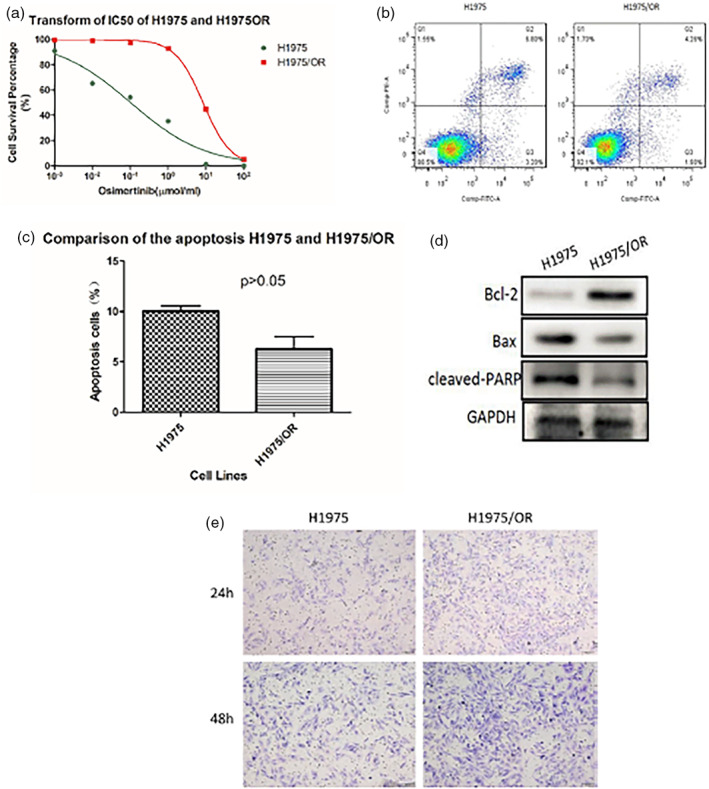
(a) IC50 of H1975 and H1975/OR. (b, c, d) Difference of apoptotic ability between H1975 and H1975OR. (e) The invasive ability of H1975 and H1975/OR by transwell assay

The authors apologize for the error and any inconvenience it may have caused.

## References

[1] Qin Q , Li X , Liang X , Zeng L , Wang J , Sun L , et al. Targeting the EMT transcription factor snail overcomes resistance to osimertinib in EGFR‐mutant non‐small cell lung cancer. Thorac Cancer. 2021;12:1708–15. 10.1111/1759-7714.13906 33943009 PMC8169301

